# Thermal Vulnerability and Potential Cultivation Areas of Four Day-Neutral Strawberries in Chile: Implications for Climate Adaptation

**DOI:** 10.3390/plants14203205

**Published:** 2025-10-18

**Authors:** Angela Sierra-Almeida, Loreto V. Morales, Diego Guerrero, Rodrigo J. N. Hasbún, Luis Retamal, Adrián Garrido-Bigotes, Ítalo Tamburrino, Andrea Maruri

**Affiliations:** 1 Grupo de Ecofisiología Térmica (GET), Facultad de Ciencias Naturales y Oceanográficas, Universidad de Concepción, Concepción 4030000, Chile; loretomorales@udec.cl (L.V.M.); dguerrero2016@udec.cl (D.G.); 2 Cape Horn International Center (CHIC), Cabo de Hornos 6350000, Chile; luis.retamal@ug.uchile.cl (L.R.); italobioambiental@gmail.com (Í.T.); 3 Facultad de Ciencias Ecológicas, Universidad de Chile, Ñuñoa 7800003, Chile; 4 Laboratorio de Epigénetica Vegetal (LEV), Facultad de Ciencias Forestales, Universidad de Concepción, Concepción 4030000, Chile; rodrigohasbun@udec.cl (R.J.N.H.); adrigarrido@udec.cl (A.G.-B.); 5 Instituto de Ecología y Biodiversidad (IEB), Concepción 4030000, Chile; 6 Agrícola Llahuén, Paine 9540000, Chile; amaruri@llahuen.com

**Keywords:** day-neutral variety, electrolyte leakage, *Fragaria × ananassa*, freezing, heat waves, LT_50_, production areas, strawberry, thermal niche

## Abstract

Understanding strawberry thermal resilience is crucial for optimizing cultivation in the face of climate change. However, its thermal niche remains underexplored. We assessed the thermal vulnerability of leaves and flowers in four day-neutral strawberry varieties cultivated in Chile and evaluated potential shifts in their suitable cultivation areas under warming scenarios. Tolerance to freezing, heat (LT_50_), and Thermal Tolerance Breadth (TTB) were determined, and habitat suitability was modeled using MaxEnt under two climate change projections and time periods. Heat LT_50_ of leaves and flowers was similar across strawberry varieties, averaging 56 °C. Conversely, the average freezing LT_50_ of flowers was 12 K less negative than that of leaves across varieties. The TTB of leaves was generally broader than that of flowers, except for San Andreas, with Monterrey displaying the broadest TTB difference (14.6 K). Climatic models indicated slight southward shifts in suitable cultivation areas under warming in Chile and globally. Nevertheless, the potential for strawberry cultivation in the more southern regions will depend on the development and implementation of cultivation strategies that effectively minimize the risk of freezing damage to the flowers. This highlights the need to plan cultivation areas according to each variety’s thermal tolerance to enhance resilience and sustainability in a changing climate.

## 1. Introduction

Global temperatures are rising due to climate change, leading to increasingly unpredictable weather patterns that pose significant threats to agricultural crops [1,2,3]. In recent years, the average global temperature has increased by 0.3–0.6 °C per decade, with projections estimating a rise of 3–6 °C by the end of the 21st century [4]. Moreover, the frequency and intensity of extreme temperature events—such as frost, cold, and/or heatwaves—are also escalating worldwide, negatively affecting plant survival and productivity [4,5]. Strawberries (*Fragaria × ananassa*) rank among the five most economically important fruit crops globally, generating billions of dollars in annual revenue. Over the past 19 years, global production has increased substantially—from 4.5 to 8.8 million tons—despite a mere 31% rise in cultivated areas, reflecting a notable improvement in average yield, which increased from 14 to 19 tons per hectare [6]. Leading producing regions, notably China, the United States, and Mexico, have driven this trend. However, a recurring pattern of a single rebound year followed by four stagnant or declining years suggests that the influence of climate cycles or soil-related factors warrants further investigation [7]. Therefore, the ability of strawberry plants to withstand climate change is vital to maximizing yields and ensuring crop resilience.

Strawberry phenology is primarily governed by edaphoclimatic factors, with temperature and photoperiod being the most important [8,9,10]. Short photoperiods and low temperatures during autumn and winter induce dormancy, while growth, flowering, and fruiting initiate in spring, driven by increasing temperatures and photoperiod, while flowering declines in summer due to excessive heat [10]. Strawberry varieties exhibit variable responses to photoperiod and temperature, broadly classified into short-day and day-neutral types. Short-day varieties flower and fruit under less than 14 h of photoperiod at 9–21 °C [11,12], whereas day-neutral varieties rely solely on temperature, with a broader temperature range of 4–29 °C, allowing extended fruiting periods [10,13,14]. These differences indicate that responses to rising temperatures and increased extreme events resulting from climate change will likely vary among strawberry varieties.

Previous studies indicate that different organs of strawberries vary in their thermal tolerance, which is for survival and productivity under temperature extremes [15,16,17]. While low temperatures are essential for flowering and fruit set, exposure at inappropriate development stages can lead to crop losses [18,19]. On the one hand, chilling and freezing temperatures reduce growth, photosynthesis, and water-use efficiency while increasing oxidative stress [16,20,21,22]. They can also cause flowers with black centers, misshapen fruits, or failure to produce fruits, reduce pollen quantity, size, and germination, as well as reduce ovule viability [19,23,24]. Conversely, high temperatures are necessary for the maturation of flowers and fruits, but excessive heat can impair organ function [15,25,26]. For example, temperatures exceeding 30 °C reduce leaf area and photosynthetic activity [27,28], the number of inflorescences, flowers, and ovules [29,30], as well as fruit set and size [31,32,33]. Thus, maintaining organs within their thermal limits is essential for ensuring consistent crop yield and fruit quality throughout the plant’s lifespan.

Although strawberry leaves seem to be more tolerant of cold/freezing temperatures than flowers [34], this difference highly depends on ontogeny and variety. For example, leaves can withstand temperatures from −2.1 to −21 °C [18,35,36,37,38,39], while floral organs may sustain significant damage just below 0 to −15.1 °C [40,41,42,43,44]. At the other extreme, vegetative tissues of strawberries are typically damaged between 35 and 53 °C [31,45,46,47], and flowers between 32 and 42 °C [32,34,48,49]. Understanding the specific thermal tolerance of leaves and flowers across strawberry varieties is imperative for mitigating the impacts of extreme temperatures on global production. Characterizing the Thermal Tolerance Breadth (TTB)—the temperature range within which plants can survive, grow, and reproduce—enables researchers to inform spatial planning, optimize yields, and reduce climate-related losses [5,50]. A wide TTB means greater resilience to temperature fluctuations, facilitating adaptation to changing environments, whereas a narrow TTB indicates higher plant vulnerability [5]. Despite efforts to assess strawberry thermal vulnerability, most studies have evaluated organ-specific thermal tolerance separately, and in response to either chilling/freezing or heat stress, leading to bias and potential misestimates of this trait across strawberry varieties. This issue is particularly relevant because protocol differences, such as gradual vs. sudden exposure to target temperatures, and injury criteria (e.g., electrolyte leakage, photoinactivation) may condition TTB estimations, highlighting the need for simultaneous, standardized assessments of leaf and flower tolerance across the full temperature spectrum.

In Chile, projections indicate an increase in mean temperature of 2–3 °C by the end of the century [51]. Concurrently, reports have shown a trend of more than one heatwave per decade across latitudes from 20° to 36° S since 1980 [51,52,53]. Although rising temperatures may decrease the frequency of spring frosts in central Chile, these events are still present under all predictive scenarios, potentially causing severe impacts on the country’s agricultural production [54]. As a result, crops located within the interior and coastal drylands between 32° and 36° S latitude, including regions such as Valparaíso and Biobío, are projected to be among the most vulnerable to temperature rises [55]. Strawberry cultivation in Chile has grown markedly, increasing from 30,000 tons in 2000 to 70,000 tons in 2022 [56,57,58,59]. Currently, 59 varieties are registered in the country [58], with Albion and Monterrey day-neutral varieties being the most cultivated [60,61]. Cultivation extends from 18° S (Arica and Parinacota region) to 38° S (Araucanía region), but approximately 80% of production is concentrated between 33° and 36° S, primarily in the Metropolitan Region and coastal areas between Valparaíso and Biobío [56,57,58,59]. About 65% of Chilean strawberries are intended for domestic consumption, with the remaining 35% exported [10]. Furthermore, Chile produces strawberry plants for export and supports markets in countries with larger production volumes. Concerningly, export volumes have declined in recent seasons to around 40,000 tons, primarily due to the widespread nematode outbreak that has severely affected plant vigor and fruit yield. This situation underscores the crop’s vulnerability to both biotic and abiotic stressors and highlights the urgent necessity for strategies that promote sustainable production amid increasing environmental variability.

Within this framework, the main goals of this study were to assess the thermal vulnerability of leaves and flowers in four day-neutral strawberry varieties cultivated in Chile and to evaluate potential shifts in their suitable cultivation areas due to global warming. Specifically, we aimed (1) to determine and compare the freezing and heat tolerance, as well as the thermal tolerance breadth of these organs, and (2) to evaluate potential shifts in suitable production areas based on the ecological niche concept, as implemented through niche modeling of *Fragaria × ananassa*. By examining these attributes, this research will provide valuable insights into the challenges posed by climate change to strawberry cultivation and contribute to the development of adaptive strategies that ensure the crop’s long-term viability.

## 2. Results

### 2.1. Differences in Heat and Freezing Tolerance, and Thermal Tolerance Breadth Between Leaves and Flowers of Strawberry Varieties

**Figure 1:**
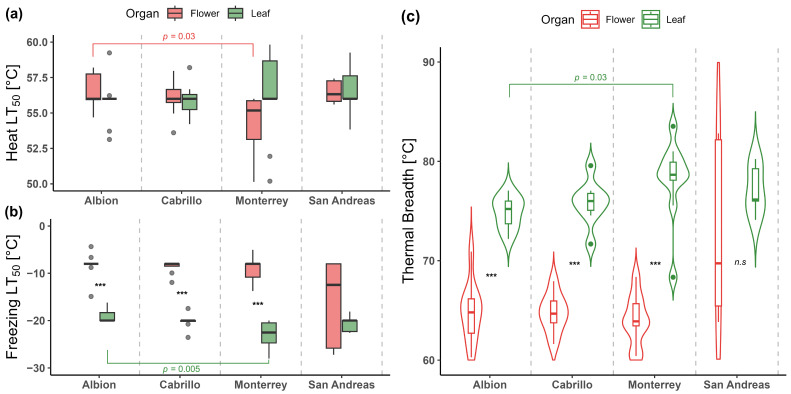
Heat (**a**), freezing (**b**) tolerance (LT_50_ °C), and thermal tolerance breadth (TTB) were determined in leaves and flowers of four strawberry varieties. Colors indicate organs: red for flowers and green for leaves. In (**a**,**b**), boxes indicate the average (=second quartile; line inside the box) and extend from the first to the third quartile. The whiskers show a maximum of 1.5-fold interquartile range. Circles represent outliers. Red and green brackets indicate significant differences in LT_50_ across varieties (*p* < 0.05). Asterisks denote significant differences between leaves and flowers (*** = *p* < 0.001). In plot (**c**), the violin represents the density of the data distribution at different variable values. Boxes inside violins indicate the median (=second quartile; line inside the box) and extend from the first to the third quartile. The whiskers show a maximum of 1.5-fold interquartile range. Circles represent outliers. The green bracket indicates significant differences in LT_50_ leaves across varieties (*p* < 0.05). Asterisks denote significant differences between leaves and flowers (*** = *p* < 0.001), n.s. represents non-significant differences.

Heat tolerance of leaves and flowers was similar for all varieties, with an LT_50_ average of 56.2 ± 0.3 °C (*Z* = 0.53, *p* = 0.566) for Albion; 55.9 ± 0.3 °C (*t* = 15, *p* = 0.902) for Cabrillo; 54.8 ± 0.7 °C (*Z* = 1.21, *p* = 0.226) for Monterrey, and 56.5 ± 0.5 °C (*t* = 8, *p* = 0.956) for San Andreas (Figure 1a; Table S1). In contrast, freezing tolerance of leaves differed considerably from that of flowers in three out of four strawberry varieties studied (Figure 1b; Table S1). Hence, the flowers of Albion were 10.7 K less tolerant to freezing than their leaves (*Z* = −3.58, *p* < 0.001). This LT_50_ difference between leaves and flowers was 11 K for Cabrillo (*Z* = −3.36, *p* < 0.001) and 13.7 K for Monterrey (*t* = 11.7, *p* < 0.0001). Although an apparent LT_50_ difference of 4.3 K was observed in San Andreas, it was not significant (*t* = −0.99; *p* = 0.351) because of high data variability observed for flowers LT_50_ (Figure 1b; Table S1). In addition, our results showed that the TTB of leaves was broader than that of flowers in three out of four varieties (Figure 1c; Table S1). For Albion, the TTB of leaves was 10 K broader than that of their flowers (*t* = 8.76; *p* < 0.0001). This difference in TTB between leaves and flowers was 9.2 K for Cabrillo (*t* = 10.56; *p* < 0.0001) and 14.6 K for Monterrey (*Z* = 1; *p* < 0.001). A TTB difference of 4.5 K between leaves and flowers was observed for San Andreas (Figure 1c; Table S1), but it was not significant (*Z* = 1.03, *p* = 0.333). Interestingly, TTB for leaves of Monterrey was 3 K broader than that of Albion (*H*_3,32_ = 8.98, *p* = 0.029; Figure 1c; Table S1), which would be explained by the lower freezing tolerance of Albion’s leaves (Table S1; Figure 1b). In contrast, all varieties showed similar TTB for flowers (*F*_3,12_ = 1.64, *p* = 0.233). Nevertheless, the TTB of San Andreas should be analyzed with caution due to the apparent greater freezing tolerance of its flowers, which would increase its TTB compared to other varieties (Figure 1c).

Additionally, the heat tolerance of leaves was similar across strawberry varieties (*H*_3,33_ = 0.42, *p* = 0.935; Figure 1a), with LT_50_ averaging 55.7 ± 0.3 °C. In contrast, the flowers of Albion showed a heat LT_50_ 2 °C higher than that of Monterrey (*H*_3,32_ = 9.15, *p* = 0.027), but similar to other varieties (Figure 1a; Table S1). In the case of freezing tolerance, LT_50_ leaves varied across strawberry varieties (*H*_3,32_ = 12.8, *p* = 0.005; Figure 1b), from −19 ± 0.5 °C (Albion) to −22.9 ± 0.8 °C (Monterrey) (Figure 1b). Specifically, Monterrey leaves tolerated freezing temperatures 2.9 K lower than Albion, but other varieties showed intermediate values (Table S1). In contrast, freezing tolerance of flowers was similar across strawberry varieties, averaging LT_50_ of −8.7 °C excluding San Andreas and of −10.6 °C including it (*H*_3,32_ = 4.62, *p* = 0.202; Figure 1b). However, as mentioned above, freezing LT_50_ of the flowers of San Andreas should be treated with caution (Figure 1b).

### 2.2. Potential Shifts in Suitable Cultivation Areas Based on Niche Models

The environmental variables used to construct the species distribution model were Isothermality (Bio3), Maximum temperature of the warmest month (Bio5), Minimum temperature of the coldest month (Bio6), Precipitation of the wettest quarter (Bio16), Precipitation of the coldest quarter (Bio19), and Elevation (Elev) (Figure S1). The relative contribution of bioclimatic variables to the MaxEnt model revealed that the minimum temperature of the coldest month (Bio6) was the most influential predictor, followed by the precipitation of the wettest quarter (Bio16) (Table S2).

**Figure 2:**
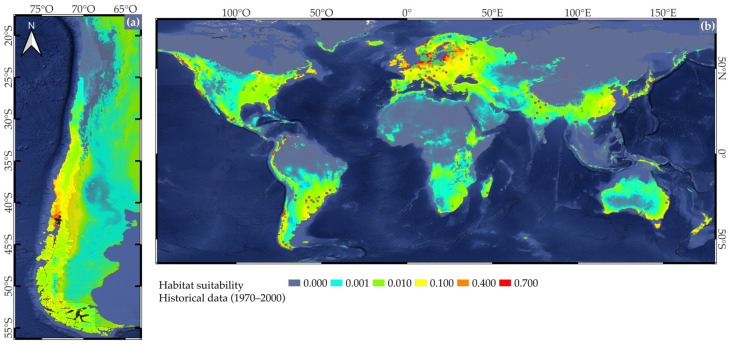
Habitat suitability for *Fragaria × ananassa* projected in the present using the selected BIO variables calculated from historical WorldClim data for (**a**) Chile and (**b**) globally. The color gradient represents suitability, with warmer colors indicating higher suitability. Green points indicate hybrid species occurrences obtained from GBIF [62] after filtering, while red points represent occurrences obtained from the literature.

According to habitat suitability projections based on historical climatic conditions (1970–2000), suitable areas for the cultivation of *Fragaria × ananassa* in Chile primarily extend between 30° and 45° S (Figure 2a), which aligns with the current production areas in the central-southern regions. However, our model also identified additional areas with high suitability that are currently underutilized for strawberry production, such as coastal and mid-elevation areas in the Araucanía and Los Ríos regions (39° to 40° S). At the global scale (Figure 2b), suitable areas for strawberry cultivation are concentrated in temperate regions of North America, Europe, and East Asia, matching the known centers of commercial production. Notably, high suitability is also observed in some parts of southern South America, southeastern Australia, and highland areas of tropical regions. These findings suggest potential opportunities for expanding or adapting cultivation areas under current climatic envelopes. The close match between high-suitability areas and known occurrence points (Figure 2) supports the robustness of the model and the relevance of thermal and precipitation-related variables in defining the ecological niche of cultivated strawberries (Figure S1).

**Figure 3:**
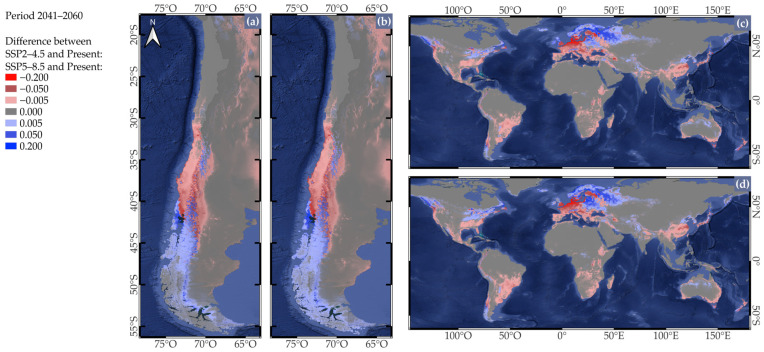
Projected changes in habitat suitability for *Fragaria* × *ananassa* based on the difference between the 2021–2040 period and the present, using selected bioclimatic variables from WorldClim and the GCM MPI-ESM1-2HR, for (**a**) Chile under the SSP2-4.5 and (**b**) SSP5-8.5 scenario; (**c**) the global scale under the SSP2-4.5 and (**d**) the SSP5-8.5 scenario. Red colors indicate a decrease (negative difference) in habitat suitability, while blue colors indicate an increase (positive difference) between the two time periods.

Habitat suitability was projected using the MPI-ESM1-2-HR model under two Shared Socioeconomic Pathways (SSPs): SSP2-4.5 and SSP5-8.5 for the period 2021–2040. For SSP2-4.5, suitable areas in Chile remain concentrated between 30° and 45° S (Figure S2a), with almost imperceptible changes. Nevertheless, when we compared this projection with historical baseline (Figure 3a), a gradual southward shift is observed, with increased suitability in parts of Los Ríos (39° to 40° S) and Los Lagos (40° to 43° S) regions, and a slight decline from northern regions such as Coquimbo (29° to 32° S) and Valparaíso (32° to 33° S) to central valleys in Los Lagos (40° to 43° S). For SSP5-8.5, projected suitable areas in Chile show similar patterns to those observed under SSP2-4.5 (Figure S2b). Compared to the historical baseline, suitable areas in Chile shift southward, expanding into regions between 39° and 43° S, while declining in northern regions located between 32° to some areas at 42° S (Figure 3b). Globally, for SSP2-4.5, suitable areas for strawberry cultivation remained in temperate regions of North America, Europe, northeastern Asia, as well as in parts of southern South America, southeastern Australia, and high-altitude areas in tropical regions (Figure S2c). However, when compared to the historical baseline, habitat suitability increased toward the poles in North America, Europe, eastern Asia, and southern South America, but it decreased in subtropical regions of North America, Europe, eastern Asia, and southeastern Australia (Figure 3c). For SSP5-8.5, projected suitable areas globally show similar patterns to those observed under SSP2-4.5 (Figure S2c,d). Compared to the historical baseline, we observed an increase in habitat suitability toward the poles in North America, southern South America, Europe, and eastern Asia, along with a consistent loss of suitability in subtropical areas (Figure 3d).

**Figure 4:**
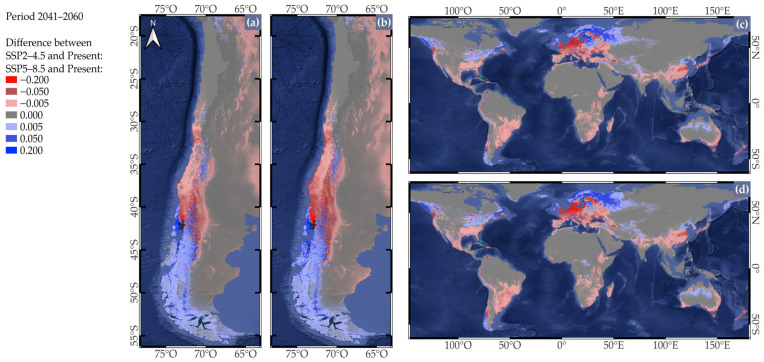
Projected changes in habitat suitability for *Fragaria × ananassa* based on the difference between the 2041–2060 period and the present, using selected bioclimatic variables from WorldClim and the GCM MPI-ESM1-2HR, for (**a**) Chile under the SSP2-4.5 and (**b**) SSP5-8.5 scenario; (**c**) the global scale under the SSP2-4.5 and (**d**) the SSP5-8.5 scenario. Red colors indicate a decrease (negative difference) in habitat suitability, while blue colors indicate an increase (positive difference) between the two time periods.

Habitat suitability projections for the 2041–2060 period under the SSP2-4.5 scenario in Chile indicated that suitable areas will continue to be located between 30° and 45° S (Figure S3a), consistent with both the current period and the 2021–2040 projections (Figure 2 and Figure S2). When comparing 2041–2060 to the historical baseline (1970–2000), a notable increase in habitat suitability was observed in Los Ríos (39° to 40° S) and Los Lagos (40° to 43° S) regions, while losses intensified in northern regions such as Coquimbo (29° to 32° S) and Valparaíso (32° to 33° S), extending southward into the central valleys of Los Lagos (40° to 43° S) (Figure 4a).

The observed southward expansion of suitable cultivation areas is consistent with the pattern previously described for the 2021–2040 period (Figure 3a). Under the SSP5-8.5 scenario for Chile, suitable areas similarly remained concentrated between 30° and 45° S (Figure S3b). Furthermore, when comparing the 2041–2060 period to the historical baseline, the trends became even more marked (Figure 4b). In this case, gains in habitat suitability extended to northern Aysén Region (44° to 45° S), while losses were particularly pronounced in the Coquimbo region (29° to 32° S), the central valleys of Los Ríos (39° to 40° S) and Los Lagos regions (40° to 43° S) and advance further south along the eastern slope of the Andes. For SSP2-4.5, globally suitable areas remained in North America, Europe, eastern Asia, southern South America, southeastern Australia, and high-elevation tropical regions (Figure S3c). Additionally, when comparing this period to the historical baseline, the pattern of increased habitat suitability toward polar regions and losses in subtropical regions was consistent with the trends observed for the 2021–2040 period (Figure 4c). Globally, suitable areas for cultivation were similar to those projected for the 2021–2040 period, regardless of SSPs (Figure 3), which were consistent with projected warming trends, where suitable cultivation areas persist in North America, Europe, eastern Asia, southern South America, as well as Australia, and high-elevation tropical regions. Once again, when comparing this period to the historical baseline, the observed increase in suitable cultivation areas toward polar regions and the loss in subtropical areas remained consistent with previous projections (Figure 4d).

## 3. Discussion

Understanding the thermal tolerance of crops like strawberries is crucial for ensuring productivity under climate change. Thus, our purpose was to elucidate the thermal tolerance of strawberry leaves and flowers across four neutral-day varieties, and to predict shifts in suitable cultivation areas under global warming scenarios. Our findings reveal that strawberry leaves and flowers exhibited distinct thermal tolerances, particularly to freezing, while heat tolerance was consistent across varieties. Niche models identified underutilized cultivation areas in Chile and globally. Additionally, they predicted a slight southward shift, with a reduction in current suitable areas for cultivation as temperatures rise. These findings not only underscore the intricate nature of plant responses to temperature but also highlight the potential of developing cultivar-specific methods to enhance strawberry resilience in the face of climate change in Chile and other regions.

The LT_50_ data for both heat and freezing exposures indicate that the strawberry varieties studied exhibit high thermal tolerance in both leaves and flowers. Specifically, leaves can withstand up to 56 °C, placing them at the upper end of the previously reported range of 39–53 °C [45,46,47]. On the other hand, flowers are generally damaged at 35–42 °C, especially near anthesis when they are most vulnerable [31,34]. However, in our varieties, flowers reached LT_50_ values up to 56 °C, indicating an unexpectedly high heat tolerance. For freezing tolerance, leaves showed an average LT_50_ of −21 °C, consistent with the upper known range of −15 to −25 °C [63,64]. Flowers, usually more vulnerable to freezing, have LT_50_ ranging from −2.1 to −7 °C [41,42,43,44], but in our study, the flowers of three varieties reached around −8.7 °C, with San Andreas standing out at −16.3 °C. The discrepancies between previously published LT_50_ values and those in our study, particularly for flowers, may be partly attributable to variety-specific traits such as morphology, sugar content, and antioxidant levels [14,30,65,66]. Stress-related metabolic responses, including the synthesis of heat-shock proteins, may also influence tolerance levels [29,67,68]. Variations in cultivation practices, such as microclimate, substrate, and irrigation, could contribute to these differences, though all varieties in this study were grown under the same conditions. Methodological differences, including thermal exposure protocols (e.g., stepwise vs. heat/freeze-shock), tissue damage assessment criteria (e.g., electrolyte leakage, Fv/Fm changes, visual injury), and development stages, may further explain discrepancies across studies. Together, these findings highlight the need for standardized protocols and further research into the epigenetic, molecular, and physiological mechanisms underlying thermal resilience in strawberry varieties.

The disparity in freezing tolerance between organs is likely due to the structural and metabolic adaptations in leaves that provide enhanced protection against cellular damage during freezing [69,70,71,72]. Conversely, flowers, with their higher water content and metabolic activity during anthesis [41,44,73], are particularly vulnerable to freezing-induced damage [74,75]. Interestingly, the absence of significant differences in freezing tolerance between leaves and flowers of the San Andreas variety indicates potential variability in how flower tissues might respond. This suggests that additional research with greater replication is needed. Nevertheless, this could imply that flowers may have higher cold hardiness. Notably, San Andreas is known to produce fruits early [76] and perform better than varieties like Albion regardless of temperature [77]. This suggests that its flowering phase may coincide with spring freeze events, where cold hardiness is crucial. Future research, including histological or gene expression studies, could shed light on the mechanisms behind this behavior.

Thermal tolerance breadth (TTB), as an integrative measure of stress tolerance, indicates the sensitivity of plants to temperature fluctuations [5]. In our study, TTB of leaves exceeded that of flowers by more than 10 °C (Figure 1; Table S1). The regions where over 80% of Chilean strawberry production occurs, between 33° and 36° S [56,57,58,59], experience historical temperature fluctuations from −4.6 to 34.4 °C in October, and from −4.7 to 41.5 °C in January (Table S3). These months correspond to periods when leaves (October) and flowers (January) are more active and abundant across varieties. The temperature fluctuations during both periods were 39 K and 46.2 K, respectively, which are significantly below the TTB obtained for both leaves and flowers (all above 63 K) of our varieties. This suggests that, despite differences in freezing tolerance between organs, all varieties possess a thermal safety margin that could enable them to withstand temperature extremes without suffering damage. The thermal safety margin reflects how close plants are to their thermal limits and their vulnerability to future warming and extreme events [78,79,80]. It is important to set out that historical temperature records were obtained from macroclimatic sources (e.g., weather stations, satellites), which may underestimate the extremity of conditions at ground level, the microenvironment where strawberries are commonly cultivated, and where temperature variability and extremes tend to be higher [74,81,82]. Such unpredictable temperature events can occur during critical periods of high crop sensitivity, potentially causing unforeseen damage to production. Moreover, given that neutral-day varieties flower earlier than short-day ones [10,11,12], they may be more freezing-tolerant, which could be advantageous for cultivation in colder areas. These findings reinforce the need for frost protection strategies—such as passive row covers, overhead irrigation, or variety selection—particularly in regions with early-season production.

If we consider the projections of suitable cultivation areas for strawberries in Chile and globally based on historical records, there is a strong consistency between the current production zones and the potential areas. However, some regions to the south of Chile—primarily along the coast and at medium altitudes in La Araucanía and Los Ríos—remain underutilized. The reasons could be associated with sociopolitical conflicts and/or other commercial activities like the forest industry occupying these areas. Nevertheless, these areas present promising opportunities where the feasibility of strawberry cultivation could be evaluated, considering the physiological tolerance of the varieties studied in this work. On the other hand, although the projected changes in habitat suitability under future scenarios are subtle, there is a consistent trend toward a southward shift, with increased availability of cooler areas for the cultivation of *Fragaria × ananassa*. In Chile, the southward shift reaches 45° S latitude. For this area, historical temperature data reported absolute minimum temperatures of −9.1 °C and −5.9 °C during October and January, respectively (Table S3). Freezing temperatures during October are particularly critical for flowers, considering that 3 out of four varieties studied exhibited an LT_50_ averaging −8.7 °C, which suggests a narrow temperature safety margin from freezing damage. At the other extreme, the heat tolerance of the leaves and flowers of our varieties is well above the absolute maximum temperatures recorded in summer in current and potential new areas for cultivation (Tables S1 and S3). However, it is important to recognize that, while extreme temperatures may not induce immediate tissue damage—particularly flower loss—exposure to heat or freezing temperatures can adversely affect the resulting fruit yield, size, and quality [19,29,32,83]. These effects highlight the significance of considering sub-lethal thermal stress in evaluating crop performance and adaptation strategies under changing climate conditions.

A decrease in suitability is projected for central Chile and subtropical regions globally, which is consistent with previous studies. For example, a recent study modeled the potential distribution’s change of 61 pollinator-dependent crop species worldwide under the SSP5-8.5 scenario for the year 2070 using MaxEnt [84]. In the case of *Fragaria × ananassa*, the study documented declines of 20.9% in Europe and 28.6% in South America, along with an increase of 73.4% in North America [84]. Moreover, the authors reported that, on average, among the 61 crop-dependent species examined, there is a projected loss of habitat suitability in South America of 14.4% by 2070, making it the second most affected continent after Australia (26.2%) [84]. On the other hand, a more specific study on strawberries at the global scale with a focus on Brazil [3] reported a shift toward colder latitudes under climate change scenarios (2050 and 2100), using CLIMEX and occurrence records from GBIF and the Brazilian Institute of Statistics and Geography (IBGE) derived from the 2017 Agricultural Census [3]. The fact that, when using MaxEnt and occurrence records from other sources, convergent results are obtained highlights the consistency of the trend in habitat suitability shifts for strawberries.

Among the climatic variables, the minimum temperature of the coldest month (Bio6) was the strongest contributor to model performance, which is consistent with the species’ physiology and with previous studies emphasizing the dominant role of temperature in strawberry distribution [3]. This suggests that the potential global distribution is largely determined by the species’ thermal limits, particularly its freezing tolerance. Similar studies have reported positive correlations between plant thermal tolerance and the maximum temperature of the warmest month [78,85] and the annual mean maximum temperature [86]. However, as a cultivated species, its distribution is not constrained by dispersal or biotic interactions; instead, agronomic practices such as irrigation, frost protection, or greenhouse cultivation can artificially expand its realized niche. For example, tunnel use increases plant survival from freezing damage but may also raise heat stress, causing flower abortion [83]. Although the models adequately capture climatic suitability under open-field conditions, extrapolations to marginal areas or highly technological production systems should be made cautiously. These findings highlight the need to explore further southward expansion options, considering the variety’s physiological constraints, and underscore the importance of developing innovative, sustainable cultivation strategies to promote both economic viability and environmental sustainability.

Although our study primarily focused on thermal extremes, it is crucial to consider interactions with other stressors, especially water availability, since precipitation was the second factor in explaining suitable cultivation areas for strawberries (Bio16; Table S2). Drought stress can exacerbate heat and freezing damage by reducing tissue hydration and membrane stability [39,87,88], which is particularly relevant given the high water dependence of strawberry cultivations. This emphasizes the urgent need for adaptive strategies, such as selecting and deploying varieties with increased tolerance to thermal extremes, optimizing planting schedules to avoid peak stress periods, and implementing soilless or substrate-based cultivation systems to better manage the root zone. The organ-specific thermal tolerance of the studied strawberry varieties supports their potential for productive cultivation across several regions in Chile and globally. To translate physiological data into practical solutions, integrating models like CROPGRO-Strawberry within DSSAT, which integrates temperature thresholds, assimilate partitioning, and environmental inputs to simulate growth and yield, becomes essential [89]. Similarly, crop models like DSSAT and SUNFLO have been instrumental in evaluating varietal performance under variable conditions to inform management strategies [90]. Our findings on organ-specific thermal tolerance and projected shifts in suitable cultivation areas can guide targeted recommendations, including selecting varieties with flower freezing tolerance for southern expansion, adjusting planting schedules in warmer regions, and employing substrate-based systems to buffer climatic stresses.

In conclusion, our work dealing with the thermal vulnerability of strawberry cultivation informs three findings: (1) flowers exhibit lower freezing tolerance than leaves, while heat tolerance remained consistent between organs across examined varieties. This functional disparity suggests that freeze-induced damage may drive cultivar selection under colder conditions more strongly than heat stress responses; (2) identified underutilized cultivation areas in Chile’s La Araucanía and Aysén regions provide immediate relocation opportunities; (3) the projected latitudinal shift in suitability necessitates revised breeding targets for photoperiod adaptation in new geographic contexts. These results advocate for policy-supported transitions to cold-adapted cultivars in traditional growing regions while leveraging Chile’s emerging suitability to buffer global production losses.

## 4. Materials and Methods

### 4.1. Plant Material

The strawberry, *Fragaria × ananassa* Duch., is a hybrid belonging to the Rosaceae family, resulting from the crossing of *Fragaria virginiana* Duch. and *Fragaria chiloensis* Linn. It is a perennial herb, stoloniferous, and with a relatively brief productive lifespan. The stem is very short, semi-subterranean, and is referred to as the crown, from which roots, stolons, leaves, and inflorescences develop. The root system is primarily concentrated within the first 30 cm. The leaves are trifoliate, with oval, serrated leaflets that are deep green on the upper side, pubescent, and gray on the underside. The flowers have white petals, numbering 5 to 6, with 20–35 stamens and a variable number of pistils. They are hermaphroditic and autogamous and may be arranged in cyme-type inflorescences [91].

**Figure 5:**
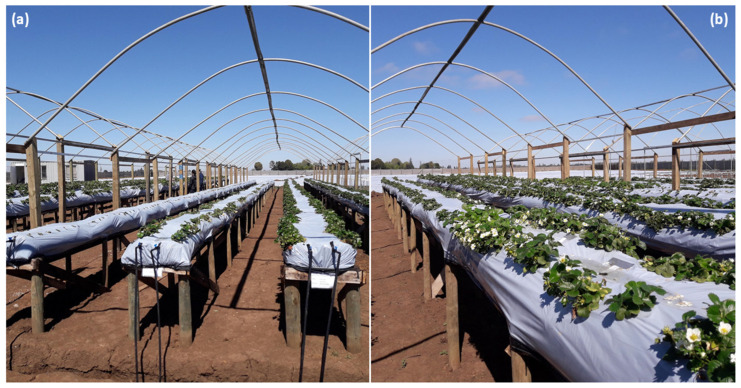
The elevated soilless system for strawberry cultivation at the Llahuén Experimental Center. (**a**) A general overview of the wooden modules where the plants were cultivated, highlighting the cover, irrigation system, and variety identifiers. The (**b**) panel shows the spacing between plants within each wooden module. These photographs are referential and do not represent the phenological state of the plants at the time of determining thermal tolerances. (Credits: A. Sierra-Almeida).

We studied day-neutral varieties, which are characterized by not having a physiological response to photoperiod; that is, they only require appropriate temperatures to induce the production of floral buds [13,14]. Selected varieties were Albion, Cabrillo, Monterrey, and San Andreas, which were produced by the Experimental Center of Agrícola Llahuén in Parral, Chile (36° S, 71° W). Albion produces intermediate-sized plants with slow initial growth at low temperatures in spring; it is a variety widely used in the fresh market and frozen agro-industry, as it accumulates a large amount of sugar in its fruits [92]. Cabrillo produces medium-sized plants that require soil temperatures above 12 °C for establishment. Flowering induction occurs at 20–25 °C, and its productive behavior is like Albion if soil temperatures do not drop below 12 °C. Monterrey is a variety with more abundant flowering than Albion. It is suitable for the fresh and frozen markets as it produces fruits with outstanding sweetness. The plants are larger and have rapid initial growth, so they should be planted at appropriate temperatures (i.e., soil >12 °C), as planting them in very cold conditions can lead to excessive vigor [92]. San Andreas is a variety with a later production onset than Albion, which is advantageous for fruit production under forced cultivation. It is appealing for the fresh market and frozen agro-industry because it is the variety that exhibits the largest size and uniformity of fruits. The plants are of intermediate size, with rapid initial vegetative growth, so they should be planted at soil temperatures above 12 °C, as they exhibit excessive vigor and a longer vegetative period in cold conditions [92]. The cultivation was carried out in an elevated soilless system, utilizing raised grow bags supported on wooden structures inside a protected tunnel (wooden modules; Figure 5).

Each 1-meter-long grow bag contained six plants, arranged in two rows of three plants each, with approximately 20 cm of spacing between individual plants within rows. The inter-row spacing within each double row was approximately 25–30 cm, and the distance between adjacent beds was around 1.1 m, allowing easy access for management and harvest. The substrate consisted of a 1:1 mixture of coconut coir and pine bark, supplied by Berry Compost, with a volume of 35 L per meter. This substrate is known for its high porosity, water retention, and good root aeration. This configuration resulted in an approximate planting density of 90,000 plants per hectare, based on the use of 15,000 linear meters of grow bags and a bed spacing of approximately 1.1 m center-to-center, as documented in the setup. Each wooden structure housed plants of a single variety. Fertilization was applied through fertigation, directly into the compost-based substrate using the irrigation system. Nutrient requirements were adjusted weekly according to the phenological stage of the crop: Vegetative (units/ha/week): N: 2.5–3.5; P_2_O_5_: 5.5–7.5; K_2_O: 3.0–4.0; CaO: 1.8–2.5; MgO: 0.8–1.0; S: 0.8–1.0; B: 0.06–0.08; ZnO: 0.12–0.16; NO_3_^−^ as % of total N: 40–50%. Reproductive stage (units/ha/week): N: 3.0–4.0; P_2_O_5_: 1.5–2.0; K_2_O: 6.0–8.0; CaO: 1.5–2.0; MgO: 0.8–1.0; S: 0.6–0.8; B: 0.04–0.05; ZnO: 0.06–0.08; NO_3_^−^ as % of total N: 70–80%. This fertigation strategy ensured optimal nutrient availability throughout both the vegetative growth and fruit production stages. The air temperature and humidity conditions during the growing period for strawberries near the Experimental Center are detailed in Table 1.

Plant samples were collected on 15 November 2021, from individual plants selected haphazardly, and 81 days after plantation. The numbers of individuals per variety were Albion (*n* = 9), Cabrillo (*n* = 8), Monterrey (*n* = 10), and San Andreas (*n* = 5). Differences in biological replications were due to the variable availability of plants with flowers. Leaves and flowers were collected at the same developmental stage within the organ. For vegetative structures, the youngest, healthiest, and fully expanded leaves were collected. For reproductive structures, flowers were collected during anthesis. Technical replicates corresponded to six flowers and nine leaves, which were collected from each individual plant; each one was covered with a moist paper towel and immediately placed inside a plastic-sealed bag. In cases where some individuals did not have enough flowers, the plant samples were completed with tissue obtained from the closest individual. Plant samples were stored inside a cooler to avoid changes in the tissue’s water status and were then transported to GET’s Lab at the University of Concepción. Within 5 h of collection, all plant samples were transferred to a refrigerator at 4 °C until thermal treatments started.

### 4.2. Protocol for Determining Thermal Tolerance in Strawberries

We designed a protocol for assessing freezing and heat tolerance based on the prevailing thermal conditions found in the cultivation areas of *Fragaria × ananassa* in Chile. For this, we reviewed historical temperature records spanning from the Metropolitan to the Biobío Region. Temperature records for these areas were obtained from the INIA agrometeorological network (https://agrometeorologia.cl/ (accessed on 23 October 2021), considering the period of the year when the plants are active (October to March). Specifically, climatic information was reviewed for five productive strawberry zones where meteorological stations exist: San Pedro de Melipilla (San Pedro) in the Metropolitan Region, Chanco (Chanco), Lomas (Pelluhue), Monteflor-Tucapel (Parral) in the Maule Region, and Human (Los Ángeles) in the Biobío Region. Depending on the location, temperature data were examined from the last 7–11 years. This information allowed us to identify the daily maximum (T_max_) and minimum (T_min_) temperatures recorded in each locality, as well as their daily fluctuations to reach extremes. For example, the highest T_max_ recorded in Los Ángeles was 42.1 °C on 26 January 2017, while the lowest T_min_ was −1.7 °C on 1 October 2018, for the same area, although T_min_ of −4.6 and −4.7 were recorded in Lo Prado (Metropolitan Region) on 31 October 2023 and on 6 January 2024, respectively (Table S3). With this information, the temperature range used to determine the thermal tolerance of strawberry plants was as follows.

For heat tolerance, five treatments were selected: 24 °C, 32 °C, 40 °C, 48 °C, and 56 °C. For freezing tolerance, previous data indicate that flowers are more sensitive to cold/freezing than leaves. Thus, different temperature sets were used. For leaves, we used 8 thermal treatments: 8 °C, 4 °C, 0 °C, −4 °C, −8 °C, −12 °C, −16 °C, and −20 °C, while for flowers, we used five treatments: 8 °C, 4 °C, 0 °C, −4 °C, and −8 °C. Additionally, we explored daily temperature fluctuations to define the warming and cooling rates of plant organs for the respective thermal tolerance protocols. The daily temperature fluctuations were discrete, with average drops of 1.4 °C h^−1^ and rises of 2.8 °C h^−1^ across the five considered zones. However, we considered that temperature fluctuations are greater closer to the ground [74]. This means that the daily temperature fluctuations obtained from meteorological stations, which are typically installed 2 m above ground, may underestimate those occurring 30 cm above ground, where strawberry plants are commonly cultivated. Therefore, warming and cooling rates of the plant tissues considered for the protocol increased to 2 and 4 °C h^−1^, respectively. Hence, freezing simulations started at 8 °C, at a cooling rate of 2 °C h^−1^ to reach the target temperatures. Plant samples were kept for two hours at each target temperature and were then returned to 8 °C at the same cooling rate. Heat simulations started at 20 °C, with a temperature increase and decrease of 4 °C h^−1^, and were then returned to 20 °C. For conducting these thermal simulations, leaves and flowers were cut near the base of the petiole and pedicel, respectively. Then, they were placed inside hermetically sealed Falcon tubes (15 mL) and put inside two cryostats (F34-ME, Julabo Labortechnik GmbH, Seelbach, Germany, and AP07R-40-A12E, Polyscience, Niles IL, USA) with a thermostable solution (Polycool HC-50, Polyscience, Niles, IL, USA) that runs in parallel to expose leaves and flowers to all independent thermal treatments.

### 4.3. Assessing Plant Thermal Damage

The temperature causing 50% tissue damage (LT_50_) in leaves and flowers was estimated by using the electrolyte leakage method. This approach quantifies cellular damage based on increased electrolyte (primarily K^+^) release due to loss of membrane integrity and stability [93,94]. It was selected over other methods (e.g., photoinactivation, visual scoring) because it allows for direct and comparable assessment of freezing and heat damage in different plant organs. LT_50_ in flowers was estimated using the entire functional structure, as both fertile and infertile tissues contribute to reproductive success by attracting pollinators, deterring herbivores (e.g., via the corolla), and facilitating seed production (e.g., via stamens and pistils).

After each temperature treatment, the tubes containing plant tissue samples were removed from the cryostat, and 10 mL of deionized water was added to each tube. The samples were then incubated at ambient temperature (~18 °C) on a shaker for 6 h to allow for electrolyte leakage. The solution’s electrical conductivity (EC) was measured using a conductance meter (Hanna HI 8733, Hanna Instruments, Woonsocket, RI, USA). Following the initial EC measurement, the tubes were sealed and placed in a boiling water bath for 30 min to induce maximum ion leakage, representing complete tissue damage. This boiling step ensures the detection of total electrolyte leakage and is commonly used to determine the maximum membrane damage in thermal tolerance studies [93,95,96,97].

The relative electrical conductivity (REC), an indicator of membrane electrolyte leakage, was calculated for each sample as a percentage:

(1) $$REC=\left(EC\;after\;temperature\;treatment/EC\;after\;boiling\right)*100$$

In addition, given that the manipulation of the samples and their incubation in deionized water can alter the REC, a control treatment was carried out for each variety. Falcon tubes containing tissue samples (5–10 replicates per tissue, incubated separately in 10 mL of deionized water) were incubated at ambient temperature for 10 h, the time needed to achieve the longest thermal treatment (−20 °C). After that, the same procedure used to measure the REC after thermal treatments was used to obtain the control treatment’s EC. Then, a corrected REC was calculated as follows:

Corrected REC = REC of the temperature treatment − REC of the control treatment(2)

The temperature that produced 50% damage (LT_50_) was determined by linear interpolation using the temperature that caused the highest percentage of electrolyte leakage (corrected REC) of <50% and the temperature that caused the lowest corrected REC of >50%. For treatments where 50% damage was not reached, polynomial regression models were fitted to the damage data to estimate the temperature at which 50% damage could occur. This approach was necessary when the data were in the ascending region of the damage curve. Polynomial models of different degrees (up to the fourth degree) were tested, and the model with the highest adjusted R^2^ was chosen to extrapolate the LT_50_ temperature. In addition, we reported the Thermal Tolerance Breadth (TTB), which is an indicator of a plant’s resilience to changes in its thermal environment, and it is determined as the temperature range between heat and freezing tolerance of a plant/species [5].

### 4.4. Climatic Data and Modeling

#### 4.4.1. Occurrence Data Processing and Definition of the Study Area

To estimate the locations in Chile and globally with suitable climatic conditions for the cultivation of *Fragaria × ananassa*, data were obtained from the Global Biodiversity Information Facility, GBIF [62], using the R package rgbif 3.8.1 [98]. The global occurrence records retrieved from GBIF were filtered using the CoordinateCleaner package version 3.0.1 [99], retaining only those labeled as “Preserved Specimens.” Records dated before 1970 were excluded to ensure compatibility with the climate layers. In contrast, no filtering based on record type was applied to the GBIF occurrences from Chile, due to the limited availability of presence data for this region. Because GBIF records did not adequately represent open-field cultivation sites of *Fragaria × ananassa* worldwide, a literature review was conducted to georeference such locations [100]. The resulting dataset, combining GBIF and literature-based records, was then spatially filtered to retain only one presence per grid cell to reduce spatial autocorrelation [101]. From the total occurrences, this process yielded 103 presence records obtained from the literature and 451 presence records obtained from GBIF, which were used for the models.

As no true absence data were available, pseudo-absence points were generated. The study area was defined based on the physiological limits of *Fragaria × ananassa* leaves. Specifically, a threshold was applied to bioclimatic variable Bio6—minimum temperature of the coldest month—retaining only locations where this variable did not fall below −20 °C.

#### 4.4.2. Climatic Variables and Filtering

Nineteen bioclimatic variables related to temperature and precipitation, along with elevation, were obtained from the WorldClim database [102] at a spatial resolution of 2.5 arc-minutes. To eliminate collinear variables, a UPGMA was performed using the Pearson correlation matrix among variables. To select one representative variable from each resulting cluster, the Mean Decrease Accuracy was calculated using the randomForest package version 4.7.1.2 in R [103], applying classification to differentiate presence points from pseudo-absences, following the Random Forest algorithm [104]. To minimize the effects of class imbalance and overlap inherent to presence-only data, down-sampling and 1000 classification trees were used [105]. Subsequently, to confirm that the selected variables were not collinear, the Variance Inflation Factor (VIF) was calculated using the usdm package version 2.1.7 in R [106], following standard procedures for detecting multicollinearity in ecological models [107]. The final selected variables were: Isothermality (Bio3), Maximum temperature of the warmest month (Bio5), Minimum temperature of the coldest month (Bio6), Precipitation of the wettest quarter (Bio16), Precipitation of the coldest quarter (Bio19), and Elevation (Elev). A final Random Forest model was run using the same procedure to determine the relative importance of each variable (Table S2).

#### 4.4.3. Model Construction and Configuration

The MaxEnt algorithm [108] was selected due to its proven effectiveness and widespread application in modeling the distribution of crop species [84,109,110,111,112]. The ENMeval package version 2.0.4 [113,114] was used to identify the optimal combination of feature classes and regularization multipliers to avoid overfitting. The feature combinations evaluated were as follows: “L”, “Q”, “H”, “LQ”, “LP”, “QH”, “QP”, “HP”, “LQH”, and “LQHP”, together with regularization multiplier values of 0.3, 0.7, 1.1, 1.6, 2.0, and 2.4. Model selection was based on Delta AICc, following the approach proposed by [115]. The optimal model was the QH combination with a regularization multiplier of 0.4.

To assess model performance, the random k-fold method was applied [116]. Using the selected configuration, the MaxEnt software version 3.4.3 was run to estimate habitat suitability. Ten replicates were generated, and the average raw output was selected for final analysis. The resulting habitat suitability raster was post-processed in QGIS [117] to produce cartographic outputs. Raw suitability values were rescaled and classified into discrete suitability categories to facilitate visualization and interpretation.

#### 4.4.4. Climate Change Scenarios

To project the potential habitat suitability of *Fragaria × ananassa* under future climate change scenarios, the six previously selected bioclimatic variables were retrieved from the CMIP6 global climate model MPI-ESM1-2-HR for the periods 2021–2040 and 2041–2060, under two Shared Socioeconomic Pathways (SSPs): SSP2-4.5 and SSP5-8.5. This resulted in a total of four climate projections. As reported in previous studies, the MPI-ESM1-2-HR general circulation model (GCM) was selected due to its high performance and reliability for the Chilean territory [118]. The MaxEnt algorithm was also used for future projections, applying the same configuration identified as optimal for the current climate conditions. This consistency in model settings ensured comparability across time periods and minimized methodological bias when evaluating shifts in habitat suitability. Finally, to evaluate the changes between future projections under the two considered scenarios and those based on historical data, difference maps were generated (see Supplementary Material for details). These maps represent the differences in habitat suitability between the 2021–2040 period under both scenarios and the Present (Figure S2), as well as between the 2041–2060 period under both scenarios and the Present (Figure S3). Negative values indicate greater habitat suitability in the present than in the future, which corresponds to a loss of suitable habitat, while positive values indicate lower habitat suitability in the present than in the future, which corresponds to a gain in suitable habitat.

### 4.5. Data Analysis

The estimation of LT_50_ values was based on polynomial regression models fitted using the lm() function from the stats package [119]. This approach was applied when the LT_50_ could not be directly determined from the observed data, selecting the model with the highest adjusted R^2^ to estimate the LT_50_ temperature. To compare freezing and heat tolerance between leaves and flowers within each variety, either paired *t*-tests (t.test, stats package) or Wilcoxon signed-rank tests (wilcox.test, stats package) were applied, depending on whether the normality assumption of the differences between pairs was met (shapiro.test, stats package). Specifically, for freezing tolerance, Wilcoxon signed-rank tests were used for Albion and Cabrillo, while paired *t*-tests were used for Monterrey and San Andreas. For heat tolerance, Wilcoxon signed-rank tests were used for Albion and Monterrey, while paired *t*-tests were used for Cabrillo and San Andreas. All tests were conducted independently for each variety. Differences in freezing and heat tolerance (LT_50_ values) among varieties were assessed using the Kruskal–Wallis one-way analysis of variance by ranks (kruskal.test, stats package), as the data did not meet normality assumptions. When significant differences were found, pairwise comparisons were conducted using Dunn’s test (dunn.test, dunn.test package; [120]) with Bonferroni adjustment. The comparison of Thermal Tolerance Breadth (TTB) between leaves and flowers was performed separately for each variety using the following tests: for Albion and Cabrillo, a paired *t*-test was used, and for Monterrey, a Mann–Whitney *U* test (wilcox.test, paired = FALSE; stats package) was applied as the data did not meet the normality assumption. For San Andreas, a paired *t*-test was used. The comparison of TTB among varieties was conducted separately for flowers using Welch’s ANOVA (oneway.test, var.equal = FALSE; stats package) to compare means, as the assumption of equal variances was not met, and for leaves using a Kruskal–Wallis test (kruskal.test, stats package), as the data did not meet the normality assumption. All analyses were performed in R (R Core Team, 2024; [119]), with data handling and visualization supported by the ggplot2 package [121].

## Figures and Tables

**Table 1:** Climatic conditions during the growing period at the experimental center of Agrícola Llahuén (Parral, Chile). Values correspond to the monthly average ± standard error; they are inside brackets and correspond to absolute temperatures. Data was obtained from the nearest weather station (Monte Flor-Tucapel, Parral, 36°24′ S 71°93′ W, 548 m above sea level; Red Agrometeorológica de INIA, https://agrometeorologia.cl/ (accessed on 23 October 2021).

	Time					
Variable	Oct.	Nov.	Dec.	Jan.	Feb.	Mar.
Mean air T (°C)	14.1 ± 0.5	17.2 ± 0.5	20.1 ± 0.5	20.2 ± 0.4	20.2 ± 0.4	17.5 ± 0.5
Minimum air T (°C)	6.9 ± 0.5 (2.6)	9.3 ± 0.4 (4.6)	11.8 ± 0.4 (7.8)	11.4 ± 0.4 (6)	11.1 ± 0.3 (8.4)	8.6 ± 0.4 (5.1)
^1^ Freq. T <5 °C (%)	32.3	10	0	0	0	0
Maximum air T (°C)	21.2 ± 0.7 (28.5)	25.1 ± 0.7 (31.3)	28.4 ± 0.8 (36.8)	29 ± 0.6 (34.7)	29.4 ± 0.6 (36.2)	26.4 ± 0.7 (33.1)
^2^ Freq. T >30 °C (%)	0	3.3	51.6	38.7	42.9	22.6
Mean RH (%)	70.5 ± 1	66.5 ± 1.4	62.2 ± 1.5	65.9 ± 1.2	62.3 ± 1.7	63.3 ± 1.4
Minimum RH (%)	39.2 ± 1.9	33.3 ± 1.8	31.3 ± 2.3	31.1 ± 1.7	27.5 ± 1.6	28 ± 1.4
Maximum RH (%)	94.8 ± 0.7	93.3 ± 0.9	89.8 ± 1.2	93.7 ± 0.8	90.3 ± 1.6	92.2 ± 1.2

^1^ Temperatures <5 °C are considered to trigger cold acclimation in plants [74]. ^2^ Temperatures >30 °C produce impairment of several metabolic processes in plants [74].

## Data Availability

The strawberry location data derived from the literature review included in this study are available in a repository (Mendeley Data, V2, https://doi.org/10.17632/8xd8bd4hd5.2). Further inquiries can be directed to the corresponding author. The climatic data presented in this study is available in the Supplementary Materials. These data were derived from the following resources available in the public domain: [https://agrometeorologia.cl/ (accessed on 17 July 2025); https://climatologia.meteochile.gob.cl/ (accessed on 17 July 2025); https://www.ecmwf.int/ (accessed on 22 July 2025)].

## Supplementary Materials

The following supporting information can be downloaded at: https://www.mdpi.com/article/10.3390/plants14203205/s1. Table S1. Thermal tolerance determinations in leaves and flowers of four strawberry varieties cultivated in Chile; Table S2. Relative importance of the environmental variables used, based on Mean Decrease Accuracy calculated using the Random Forest algorithm to classify *Fragaria × ananassa* presence records against pseudo-absence data; Table S3. Historical temperature records across Chilean regions where strawberry varieties are currently cultivated, as well as in the southern regions with potential for cultivation expansion; Figure S1. Climatic variables and filtering for modeling; Figure S2. Projected changes in habitat suitability for *Fragaria × ananassa* based on the difference between the 2021–2040 period and the present; Figure S3. Projected changes in habitat suitability for *Fragaria × ananassa* based on the difference between the 2041–2060 period and the present.

## Abbreviations

The following abbreviations are used in this manuscript:

EC	Electrical Conductivity
REC	Relative Electrical Conductivity
Corrected REC	Corrected Relative Electrical Conductivity
LT_50_ freezing	Freezing Temperature that Produces 50% Tissue Damage (°C)
LT_50_ heat	High Temperature that Produces 50% Tissue Damage (°C)
TTB	Thermal Tolerance Breadth
GBIF	Global Biodiversity Information Facility
MaxEnt	Maximum Entropy
SSP2-4.5	Shared Socioeconomic Pathway 2, Intermediate Development Scenario with ~4.5 W/m^2^
SSP5-8.5	Shared Socioeconomic Pathway 5, Fossil-Fueled Development Scenario with ~8.5 W/m^2^ Radiative Forcing by 2100

## References

[1] Malhi G.S., Kaur M., Kaushik P. (2021). Impact of climate change on agriculture and its mitigation strategies: A review. Sustainability.

[2] Bacelar E., Pinto T., Anjos R., Morais M.C., Oliveira I., Vilela A., Cosme F. (2024). Impacts of Climate Change and Mitigation Strategies for Some Abiotic and Biotic Constraints Influencing Fruit Growth and Quality. Plants.

[3] da Silva L.R., Araújo F.H.V., Ferreira S.R., dos Santos J.C.B., de Abreu C.M., Siqueira da Silva R., Regina da Costa M. (2024). Strawberries in a warming world: Examining the ecological niche of *Fragaria × ananassa* Duch-Across different climate scenarios. J. Berry Res..

[4] Lee H., Romero J., IPCC (2023). Climate Change 2023: Synthesis Report. Contribution of Working Groups I, II and III to the Sixth Assessment Report of the Intergovernmental Panel on Climate Change. Core Writing Team.

[5] Geange S.R., Arnold P.A., Catling A.A., Coast O., Cook A.M., Gowland K.M., Leigh A., Notarnicola R.F., Posch B.C., Venn S.E. (2021). The thermal tolerance of photosynthetic tissues: A global systematic review and agenda for future research. New Phytol..

[6] Hummer K.E., Williams K.A., Bushakra J.M., Greene S., Williams K., Khoury C., Kantar M., Marek L. (2019). North American Crop Wild Relatives of Temperate Berries (*Fragaria* L., *Ribes* L., *Rubus* L., and *Vacciniun* L.). North American Crop Wild Relatives.

[7] Cossio A.J., Flores A.A. (2021). Competitividad de la fresa mexicana en el mercado estadounidense de 1992 a 2017. Cienc. Tecnol. Agropecu..

[8] Sønsteby A., Heide O.M. (2007). Long-day control of flowering in everbearing strawberries. J. Hortic. Sci. Biotechnol..

[9] Yoneda A., Yasutake D., Hidaka K., Muztahidin N.I., Miyoshi Y., Kitano M., Okayasu T. (2020). Effects of supplemental lighting during the period of rapid fruit development on the growth, yield, and energy use efficiency in strawberry plant production. Int. Agrophys..

[10] Morales A., Vargas S. (2017). Manual de Manejo Agronómico de la Frutilla.

[11] Sønsteby A., Heide O.M. (2006). Dormancy relations and flowering of the strawberry cultivars Korona and Elsanta as influenced by photoperiod and temperature. Sci. Hortic..

[12] Heide O.M., Stavang J.A., Sønsteby A. (2013). Physiology and genetics of flowering in cultivated and wild strawberries—A review. J. Hortic. Sci. Biotechnol..

[13] Rowley D., Black B.L., Drost D., Feuz D. (2011). Late-season Strawberry Production Using Day-neutral Cultivars in High-elevation High Tunnels. Hortscience.

[14] Gude K., Stanley H., Rivard C.L., Cunningham B., Kang Q., Pliakoni E.D. (2021). Quality of day-neutral strawberries grown in a high tunnel system. Sci. Hortic..

[15] Menzel C.M. (2023). A review of fruit development in strawberry: High temperatures accelerate flower development and decrease the size of the flowers and fruit. J. Hortic. Sci. Biotechnol..

[16] Jiang N., Yang Z., Luo J., Wang C. (2023). Quantifying Chilling Injury on the Photosynthesis System of Strawberries: Insights from Photosynthetic Fluorescence Characteristics and Hyperspectral Inversion. Plants.

[17] Seymour Z.J., Mercedes J.F. (2024). Effect of heat acclimation on thermotolerance of in vitro strawberry plantlet. Folia Hortic..

[18] Maughan T.L., Black B.L., Drost D. (2015). Critical temperature for sub-lethal cold injury of strawberry leaves. Sci. Hortic..

[19] Cui M., Pham M.D., Hwang H., Chun C. (2021). Flower development and fruit malformation in strawberries after short-term exposure to high or low temperature. Sci. Hortic..

[20] Bo Y., Zhang H., Tong Y., Jia Y., Liu X., Yang L., Zuo Z., Wang Y. (2024). Growth, antioxidant enzyme activity and transcriptome response to low-temperature induction of flowering in cultivated strawberry. Plant Stress.

[21] Xu C., Wang M.T., Yang Z.Q., Wang M.T. (2020). Low temperature and low irradiation induced irreversible damage of strawberry seedlings. Photosynthetica.

[22] Roussos P.A., Ntanos E., Tsafouros A., Denaxa N.K. (2020). Strawberry physiological and biochemical responses to chilling and freezing stress and application of alleviating factors as countermeasures. J. Berry Res..

[23] Ariza M.T., Soria C., Martínez-Ferri E. (2015). Developmental stages of cultivated strawberry flowers in relation to chilling sensitivity. AoB Plants.

[24] Goswami A.K., Singh S.K., Pradhan S., Sharma R.M., Yamdagni R., Dubey A.K., Pandey V. (2019). Physiological disorders. Strawberries, Production, Postharvest Management and Protection.

[25] Menzel C.M. (2024). Temperatures above 30 °C decrease leaf growth in strawberry under global warming. J. Hortic. Sci. Biotechnol..

[26] Ullah I., Toor M.D., Yerlikaya B.A., Mohamed H.-I., Yerlikaya S., Basit A., ur Rehman A. (2024). High-temperature stress in strawberry: Understanding physiological, biochemical and molecular responses. Planta.

[27] Carlen C., Potel A.M., Ançay A. (2009). Photosynthetic response of strawberry leaves to changing temperatures. Acta Hortic..

[28] Nathewet P. (2017). Effects of heat stress on physiological index in cultivated strawberries. Khon Kaen Agric. J..

[29] Ledesma N.A., Nakata M., Sugiyama N. (2008). Effect of high temperature stress on the reproductive growth of strawberry cvs. ‘Nyoho’ and ‘Toyonoka’. Sci. Hortic..

[30] Maughan T.L. (2013). Optimizing Systems for Cold-Climate Strawberry Production. Master’s Thesis.

[31] Zini L.M., Galati B.G., Carrera C.S. (2023). High temperatures during late floral bud stages decrease fertilization in strawberry (*Fragaria × ananassa*): Pollen-pistil interaction and anatomical evidences. Plant Biosyst..

[32] Kadir S., Sidhu G., Al-Khatib K. (2006). Strawberry (*Fragaria ×·ananassa* Duch.) Growth and Productivity as Affected by Temperature. HortScience.

[33] Suzuki N., Rivero R.M., Shulaev V., Blumwald E., Mittler R. (2014). Abiotic and biotic stress combinations. New Phytol..

[34] Ledesma N.A., Kawabata S. (2016). Responses of two strawberry cultivars to severe high temperature stress at different flower development stages. Sci. Hortic..

[35] Paquin R., Bolduc R., Zizka J., Pelletier G., Lechasseur P. (1989). Tolerance au gel et teneur en sucres et en proline du collet du fraisier (*Fragaria annassa* Duch) durant L’hiver. Can. J. Plant Sci..

[36] O’Neill S.D., Priestley D.A., Chabot B.F. (1981). Temperature and Aging Effects on Leaf Membranes of a Cold Hardy Perennial, *Fragaria virginiana*. Plant Physiol..

[37] Owens C.L., Thomashow M.F., Hancock J.F., Iezzoni A.F. (2002). *CBF1* orthologs in sour cherry and the strawberry and heterologous expression of *CBF1* in strawberry. J. Am. Soc. Hortic. Sci..

[38] Turhan E., Aydoğan Ç., Akoglu A., Baykul A., Evrenosoğlu Y. (2012). Relationship of seasonal changes in antioxidative enzymes and cold hardiness in strawberry plants. J. Food Agric. Environ..

[39] Rajashekar C.B., Panda M. (2014). Water stress is a component of cold acclimation process essential for inducing full freezing tolerance in strawberry. Sci. Hortic..

[40] Sarikhani H., Safariyan-Nejad M.-S. (2021). Improving of Winter Cold Hardiness by Glycine Betaine in Strawberry. Int. J. Hortic. Sci. Technol..

[41] Ourecky D.K., Reich J.E. (1976). Frost Tolerance in Strawberry Cultivars. HortScience.

[42] Boyce B.R., Strater J.B. (1984). Comparison of frost injury in strawberry buds, blossoms and immature fruit. Adv. Strawb. Prod..

[43] Ki W.K., Warmund M.R. (1992). Low-temperature Injury to Strawberry Floral Organs at Several Stages of Development. HortScience.

[44] Hummel R.L., Moore P.P. (1997). Freeze resistance of Pacific Northwest Strawberry flowers. J. Am. Soc. Hortic. Sci..

[45] Gulen H., Eris A. (2003). Some physiological changes in strawberry (*Fragaria × ananassa* ‘Camarosa’) plants under heat stress. J. Hortic. Sci. Biotechnol..

[46] Kesici M., Gulen H., Ergin S., Turhan E., Ipek A., Koksal N. (2013). Heat-stress Tolerance of Some Strawberry (*Fragaria* × *ananassa*) Cultivars. Not. Bot. Horti Agrobot. Cluj.

[47] Ergin S., Gülen H., Kesici M., Turhan E., Ipek A., Köksal N. (2016). Effects of high temperature stress on enzymatic and nonenzymatic antioxidants and proteins in strawberry plants. Turk. J. Agric. For..

[48] Proexant (2002). El Cultivo de la Fresa.

[49] Esquivel M.A., ICAMEX (2006). Guía Técnica Para el Cultivo de la Fresa.

[50] Vitasse Y., Rebetez M. (2018). Unprecedented risk of spring frost damage in Switzerland and Germany in 2017. Clim. Change.

[51] Garreaud R. (2011). Cambio Climático: Bases Físicas e Impactos en Chile. Rev. Tierra Adentro-INIA.

[52] Pica-Téllez A., Garreaud R., Meza F., Bustos S., Falvey M., Ibarra M., Duarte K., Ormazábal R., Dittborn R., Silva I. (2020). Informe Proyecto ARClim: Atlas de Riesgos Climáticos para Chile.

[53] González-Reyes Á., Jacques-Coper M., Bravo C., Rojas M., Garreaud R. (2023). Evolution of heatwaves in Chile since 1980. Weather Clim. Extrem..

[54] Fernandez E., Whitney C., Cuneo I.F., Luedeling E. (2020). Prospects of decreasing winter chill for deciduous fruit production in Chile throughout the 21st century. Clim. Change.

[55] Villarroel C., DGAC (2023). Reporte anual de la evolución del clima en Chile. Documento elaborado por la Oficina Cambio Climático de la Sección Climatología de la Dirección Meteorológica de Chile.

[56] Asagrin (2007). Estrategias Regionales de Competitividad por Rubro. “FRUTILLAS REGIÓN METROPOLITANA”.

[57] González C. (2013). Frutillas y Moras Procesadas: La Irrupción de los Otros Berries.

[58] SAG Lista de Variedades Oficialmente Descritas. Servicio Agrícola y Ganadero (SAG). https://www.sag.gob.cl/ambitos-de-accion/lista-de-variedades-oficialmente-descritas.

[59] ODEPA *Boletín de Hortalizas*. Oficina de Estudios y políticas Agrarias (ODEPA). https://www.odepa.gob.cl/publicaciones/boletines/boletin-de-hortalizas-octubre-2024.

[60] Red Agrícola (2015). Manual del Cultivo de Frambuesas y Frutillas en Chile.

[61] Muñoz M. (2016). Evolución de los Frutales.

[62] GBIF Occurrence Download.

[63] Houde M., Dallaire S., N’Dong D., Sarhan F. (2004). Overexpression of the acidic dehydrin WCOR410 improves freezing tolerance in transgenic strawberry leaves. Plant Biotechnol. J..

[64] Zareei E., Karami F., Gholami M., Ershadi A., Avestan S., Aryal R., Gohari G., Farooq M. (2021). Physiological and biochemical responses of strawberry crown and leaf tissues to freezing stress. BMC Plant Biol..

[65] Balasooriya B.L.H.N., Dassanayake K.B., Ajlouni S. (2020). High temperature effects on strawberry fruit quality and antioxidant contents. Acta Hortic..

[66] Chamorro M.F., Mazzoni A., Lescano M.N., Fernandez A., Reiner G., Langenheim M.E., Mattera G., Robredo N., Garibaldi L., Quintero C. (2025). Wild vs. cultivated strawberries: Differential fruit quality traits and antioxidant properties in *Fragaria chiloensis* and *Fragaria* × *ananassa*. Discov. Food.

[67] Ledesma N., Kawabata S., Sugiyama N. (2004). Effect of High Temperature on Protein Expression in Strawberry Plants. Biol. Plant..

[68] Wang S.Y., Lin H.-S. (2006). Effect of plant growth temperature on membrane lipids in strawberry (*Fragaria* × *ananassa* Duch.). Sci. Hortic..

[69] Sakai A., Larcher W. (1987). Frost Survival of Plants. Responses and Adaptation to Freezing Stress.

[70] Fields P.A. (2001). Review: Protein function at thermal extremes: Balancing stability and flexibility. Comp. Biochem. Physiol. Part A.

[71] Awasthi R., Bhandari K., Nayyar H. (2015). Temperature stress and redox homeostasis in agricultural crops. Front. Environ. Sci..

[72] Arora R., Taulavuori K. (2016). Increased Risk of Freeze Damage in Woody Perennials VIS-À-VIS Climate Change: Importance of Deacclimation and Dormancy Response. Front. Environ. Sci..

[73] Jiang N., Yang Z., Zhang H., Xu J., Li C. (2023). Effect of Low Temperature on Photosynthetic Physiological Activity of Different Photoperiod Types of Strawberry Seedlings and Stress Diagnosis. Agronomy.

[74] Larcher W. (2003). Physiological Plant Ecology. Ecophysiology and Stress Physiology of Functional Groups.

[75] Yu M., Zhaxi L., Deqing Z., Wei X., Tang Y. (2025). Advances in plant response to low-temperature stress. Plant Growth Regul..

[76] Gervini Menezes F.O., Vieira Neto J. (2019). Evaluation of neutral day strawberry cultivars “Albion” and “San Andreas” under semi-hydroponic cultivation in the Alto Vale do Itajaí–SC. Rev. Thema.

[77] Hernández-Martínez N., Salazar-Gutiérrez M., Chaves-Córdoba B., Wells D., Foshee W., McWhirt A. (2023). Model Development of the Phenological Cycle from Flower to Fruit of Strawberries (*Fragaria* × *ananassa*). Agronomy.

[78] O’Sullivan O.S., Heskel M.A., Reich P.B., Tjoelker M.G., Weerasinghe L.K., Penillard A., Zhu L., Egerton J.J.G., Bloomfield K.J., Creek D. (2017). Thermal limits of leaf metabolism across biomes. Glob. Change Biol..

[79] Sastry A., Barua D. (2017). Leaf thermotolerance in tropical trees from a seasonally dry climate varies along the slow-fast resource acquisition spectrum. Sci. Rep..

[80] Kitudom N., Fauset S., Zhou Y., Fan Z., Li M., He M., Zhang S., Xu K., Lin H. (2022). Thermal safety margins of plant leaves across biomes under a heatwave. Sci. Total Environ..

[81] Curtis E.M., Gollan J., Murray B.R., Leigh A. (2016). Native microhabitats better predict tolerance to warming than latitudinal macro-climatic variables in arid-zone plants. J. Biogeogr..

[82] Körner C., Hiltbrunner E. (2018). The 90 Ways to Describe Plant Temperature. Perspect. Plant Ecol. Evol. Syst..

[83] Menzel C.M. (2025). A review of strawberry under protected cultivation: Yields are higher under tunnels than in the open field. J. Hortic. Sci. Biotechnol..

[84] Rahimi E., Jung C. (2024). A global estimation of potential climate change effects on pollinator-dependent crops. Agric. Res..

[85] Perez T.M., Feeley K.J. (2020). Photosynthetic heat tolerances and extreme leaf temperatures. Funct. Ecol..

[86] Zhu L.L., Bloomfield K.J., Hocart C.H., Egerton J.J.G., O’Sullivan O.S., Penillard A., Weerasinghe L.K., Atkin O. (2018). Plasticity of photosynthetic heat tolerance in plants adapted to thermally contrasting biomes. Plant Cell Environ..

[87] Hassan M.U., Chattha M.U., Khan I., Chattha M.B., Barbanti L., Aamer M., Iqbal M.M., Nawaz M., Mahmood A., Ali A. (2021). Heat stress in cultivated plants: Nature, impact, mechanisms, and mitigation strategies—A review. Plant Biosyst..

[88] Rawat N., Singla-Pareek S.L., Pareek A. (2021). Membrane dynamics during individual and combined abiotic stresses in plants and tools to study the same. Physiol. Plant..

[89] Yang Q., Liu L., Zhou J., Rogers M., Jin Z. (2024). Predicting the growth trajectory and yield of greenhouse strawberries based on knowledge-guided computer vision. Comput. Electron. Agric..

[90] Casadebaig P., Mestries E., Debaeke P. (2016). A model-based approach to assist variety assessment in sunflower crop. Eur. J. Agron..

[91] Villagrán V., Reyes M., Zschau B. (2012). Morfología y fisiología. Frutilla, Consideraciones Productivas y Manejo.

[92] Zschau B., Legarraga M., Reyes M., Zschau B. (2012). Variedades. Frutilla, Consideraciones Productivas y Manejo.

[93] Wilner J. (1960). Relative and absolute electrolytic conductance tests for frost hardiness of apple varieties. Can. J. Plant Sci..

[94] Lipp C.C., Goldstein G., Meinzer F.C., Niemczura W. (1994). Freezing tolerance and avoidance in high-elevation Hawaiian plants. Plant Cell Environ..

[95] Arias N.S., Bucci S.J., Scholz F.G., Goldstein G. (2015). Freezing avoidance by supercooling in *Olea europaea* cultivars: The role of apoplastic water, solute content and cell wall rigidity. Plant Cell Environ..

[96] Zhang Y.J., Bucci S.J., Arias N.S., Scholz F.G., Hao G.Y., Cao K.F., Goldstein G. (2016). Freezing resistance in Patagonian woody shrubs: The role of cell wall elasticity and stem vessel size. Tree Physiol..

[97] Morales L.V., Alvear C., Sanfuentes C., Saldaña A.O., Sierra-Almeida A. (2020). Does the life-history strategy determine the freezing resistance of flowers and leaves of alpine herbaceous species?. Alp. Bot..

[98] Chamberlain S., Barve V., Mcglinn D., Oldoni D., Desmet P., Geffert L., Ram K. (2025). rgbif: Interface to the Global Biodiversity Information Facility API.R Package Version 3.8.1. https://CRAN.R-project.org/package=rgbif.

[99] Zizka A., Silvestro D., Andermann T., Azevedo J., Ritter C.D., Edler D., Farooq H., Herdean A., Ariza M., Scharn R. (2019). Coordinate Cleaner: Standardized Cleaning of Occurrence Records from Biological Collection Databases. Methods Ecol. Evol..

[100] Sierra-Almeida A., Morales L.V., Guerrero D., Hasbún R.J.N., Retamal L., Tamburrino I., Garrido-Bigotes A. (2025). Global Records of Cultivated *Fragaria* × *ananassa* for Climate Modeling and Adaptive Management in Chile. Mendeley Data V2.

[101] Boria R.A., Olson L.E., Goodman S.M., Anderson R.P. (2014). Spatial Filtering to Reduce Sampling Bias Can Improve the Performance of Ecological Niche Models. Ecol. Model..

[102] Fick S.E., Hijmans R.J. (2017). WorldClim 2: New 1-km Spatial Resolution Climate Surfaces for Global Land Areas. Int. J. Climatol..

[103] Liaw A., Wiener M. (2002). Classification and Regression by randomForest. R News.

[104] Breiman L. (2001). Random Forests. Mach. Learn..

[105] Valavi R., Elith J., Lahoz-Monfort J.J., Guillera-Arroita G. (2021). Predictive performance of presence-only species distribution models: A benchmark study with reproducible code. Ecol. Monogr..

[106] Naimi B., Hamm N.A.S., Groen T.A., Skidmore A.K., Toxopeus A.G. (2014). Where is positional uncertainty a problem for species distribution modelling?. Ecography.

[107] Dormann C.F., Elith J., Bacher S., Buchmann C., Carl G., Carré G., García Márquez J.R., Gruber B., Lafourcade B., Leitão P.J. (2013). Collinearity: A review of methods to deal with it and a simulation study evaluating their performance. Ecography.

[108] Phillips S.J., Anderson R.P., Schapire R.E. (2006). Maximum Entropy Modeling of Species Geographic Distributions. Ecol. Model..

[109] Lin Z., Chen C., Liu Y., Liu G., He P., Liao G., Shao Z. (2022). Simulation of Citrus Production Space Based on MaxEnt. Front. Environ. Sci..

[110] Fitzgibbon A., Pisut D., Fleisher D. (2022). Evaluation of Maximum Entropy (Maxent) Machine Learning Model to Assess Relationships between Climate and Corn Suitability. Land.

[111] Ali S., Makanda T.A., Umair M., Ni J. (2023). MaxEnt Model Strategies to Studying Current and Future Potential Land Suitability Dynamics of Wheat, Soybean and Rice Cultivation under Climatic Change Scenarios in East Asia. PLoS ONE.

[112] Ali S., Umair M., Makanda T.A., Shi S., Hussain S.A., Ni J. (2024). Modeling Current and Future Potential Land Distribution Dynamics of Wheat, Rice, and Maize under Climate Change Scenarios Using MaxEnt. Land.

[113] Muscarella R., Galante P.J., Soley-Guardia M., Boria R.A., Kass J.M., Uriarte M., Anderson R.P. (2014). ENMeval: An R package for conducting spatially independent evaluations and estimating optimal model complexity for Maxent ecological niche models. Methods Ecol. Evol..

[114] Kass J.M., Muscarella R., Galante P.J., Boria R.A., Soley-Guardia M., Anderson R.P. (2021). ENMeval 2.0: Redesigned for customizable and reproducible modeling of species’ niches and distributions. Methods Ecol. Evol..

[115] Warren D.L., Seifert S.N. (2011). Ecological niche modeling in Maxent: The importance of model complexity and the performance of model selection criteria. Ecol. Appl..

[116] Hastie T., Tibshirani R., Friedman J. (2009). The Elements of Statistical Learning: Data Mining, Inference, and Prediction.

[117] QGIS Development Team (2024). *QGIS Geographic Information System. Version 3.40.5 “Bratislava”*. Open Source Geospatial Foundation Project. http://qgis.org.

[118] Gateño F., Mendoza P.A., Vásquez N., Lagos-Zúñiga M., Jiménez H., Jerez C., Montserrat S. (2024). Screening CMIP6 Models for Chile Based on Past Performance and Code Genealogy. Clim. Change.

[119] R Core Team (2024). R: A Language and Environment for Statistical Computing.

[120] Dinno A. (2017). dunn.test: Dunn’s Test of Multiple Comparisons Using Rank Sums. https://cran.r-project.org/web/packages/dunn.test/dunn.test.pdf.

[121] Wickham H. (2016). ggplot2: Elegant Graphics for Data Analysis.

