# Supplementary material for: Thermal Vulnerability and Potential Cultivation Areas of Four Day-Neutral Strawberries in Chile: Implications for Climate Adaptation
Source: Plants (Basel). 2025 Oct 18;14(20):3205. doi: 10.3390/plants14203205 (PMC12566917; doi:10.3390/plants14203205)
Supplement: Supplementary file 1 [file plants-14-03205-s001.zip › plants-3908755-supplementary.pdf]

SUPPLEMENTARY MATERIAL

Sierra-Almeida et al. Thermal Vulnerability and Potential Cultivation areas of four Day-Neutral Strawberries in Chile: Implications for Climate Adaptation

Table S1. Thermal tolerance determinations in leaves (L) and flowers (F) of four strawberry varieties cultivated in Chile. Values of heat and freezing tolerance (LT_{50}, °C), and Thermal Tolerance Breadth (TTB, °C) are shown as mean ± standard error. TTB corresponds to the temperature range between estimated freezing and heat LT_{50}. Different uppercase letters represent significant differences between plant organs (p <0.05).

	Heat LT_{50}		***Freezing*** LT_{50}		***TTB***	
Variety	**L**	**F**	**L**	**F**	**L**	**F**
Albion	55.81 ± 0.54^{a}	56.58 ± 0.38^{a}	-19.04 ± 0.43^{a}	-8.3 ± 0.88^{b}	74.85 ± 0.5^{a}	64.88 ± 0.95^{b}
Cabrillo	55.91 ± 0.32^{a}	55.98 ± 0.40^{a}	-19.74 ± 0.32^{a}	-8.73 ± 0.46^{b}	73.88 ± 1.96^{a}	64.72 ± 0.61^{b}
Monterrey	55.26 ± 1.18^{a}	54.36 ± 0.6^{a}	-22.94 ± 0.8^{a}	-9.24 ± 0.77^{b}	78.19 ± 1.21^{a}	63.61 ± 0.82^{b}
San Andreas	56.54 ± 0.91^{a}	56.49 ± 0.37^{a}	-20.61 ± 0.83^{a}	-16.31 ± 4.26^{a}	77.15 ± 1.13^{a}	72.79 ± 4.08^{a}

Table S2. Relative importance of the environmental variables used, based on Mean Decrease Accuracy calculated using the Random Forest algorithm to classify *Fragaria × ananassa* presence records against pseudo-absence data. Higher values indicate a greater contribution to model performance. See Materials and Methods for model settings.

**Variable**	**Mean Decrease Accuracy**
Min Temperature of Coldest Month (Bio6)	42.914990
Precipitation of Wettest Quarter (Bio16)	36.792279
Isothermality (BIO2/BIO7) (×100) (Bio3)	28.015789
Max Temperature of Warmest Month (Bio5)	18.162506
Elevation (Elev)	12.279205
Precipitation of Coldest Quarter (Bio19)	6.553863

Table S3. Historical temperature records across Chilean regions where strawberry varieties are currently cultivated, as well as in the southern regions with potential for cultivation expansion. The temperature data correspond to the absolute minimum (Abs T_{min}, °C) and absolute maximum temperature (Abs T_{max}, °C) recorded in October and January by 22 weather stations, combined with Eras5 data. The data span from 1914 to 2025, depending on the source and locality. Complete information is free available from the climatic services of the Dirección Meteorológica de Chile (https://climatologia.meteochile.gob.cl/) and ERA5 from the European Centre for Medium-Range Weather Forecasts (ECMWF) (https://www.ecmwf.int/).

					October		*January*	
Weather station	**Latitude**	**Longitude**	**Elevation (m asl)**	**location**	**Abs T_{min} (°C)**	**Abs T_{max} (°C)**	**Abs T_{min} (°C)**	**Abs T_{max} (°C)**
Viña del Mar Ad. Torquemada	32° 57′ 58′′	71° 29′ 34′′	140	coastal	3.4 (10/2/2020)	34.2 (10/8/2019)	7.4 (01/19/2022)	33.8 (01/17/2017)
Rodelillo, Ad.	33° 4′ 55′′	71° 33′ 23′′	335	coastal	-3 (10/11/2023)	32.3 (10/8/2019)	7.4 (01/22/2022)	34.6 (01/29/2003)
Eulogio Sánchez,Tobalaba Ad.	33° 27′ 19′′	70° 33′ 55′′	649	foothills	-2 (10/3/2021)	34.4 (10/28/24)	6.8 (01/17/2022)	37.4 (01/26/2019)
Quinta Normal, Santiago	33° 27′ 42′′	70° 28′ 0.4′′	520	valley	-4.3 (10/10/2021)	34.3 (10/28/2024)	5.9 (01/17/2022)	38.3 (01/26/2019)
Lo Prado Cerro San Francisco	33° 28′ 29′′	70° 56′ 55′′	1068	foothills	-4.6 (10/31/2023)	30.8 (10/25/2021)	-4.7 (01/6/2024)	36.8 (01/09/2024)
El Paico	33° 42′ 23′′	71° 1′ 29′′	275	valley	-0.1 (10/4/2019)	33.9 (10/8/2019)	5.8 (01/20/2022)	37.6 (01/20/2017)
Santo Domingo Ad	33° 39′ 22′′	71° 37′ 48′′	77	coastal	-1.2 (10/11/2023)	29.7 (10/18/1977)	3 (01/29/1986)	33.7 (01/29/2003)
General Freire, Ad.	34° 58′ 10′′	71° 13′ 1′′	220	valley	-4.2 (10/15/2021)	31.7 (10/31/2022)	-0.8 (01/18/2022)	37.3 (01/25/2017)
General Bdo. O'Higgins, Chillán Ad.	36° 35′ 9′′	72° 2′ 12′′	152	valley	-2 (10/24/1974)	31.9 (10/27/2016)	1.8 (01/18/2022)	41.5 (01/26/2017)
Carriel Sur, Ap.	36° 47′ 50′′	73° 4′ 1′′	8	coastal	-0.8 (10/5/1990)	27.6 (10/31/1983)	4.6 (01/20/1976)	34.1 (01/26/2017)
María Dolores, Los Ángeles Ad.	37° 24′ 49′′	72° 25′ 26′′	114	valley	-3.5 (10/15/2022)	31.5 (10/31/2020)	0.4 (01/18/2022)	42.1 (01/26/2017)

La Araucanía, Ad-	38° 56′ 4′′	72° 39′ 12′′	98	valley	-1.5 (10/8/2022)	28.8 (10/27/2016)	0.3 (01/18/2022)	34.7 (01/26/2017)
Estación Pichoy, Ad.	39° 39′ 24′′	73° 5′ 14′′	18	valley	-3 (10/7/2021)	29.2 (10/30/1999)	-0.7 (01/18/2022)	35.2 (01/26/2017)
Estación Cañal Bajo, Osorno Ad.	40° 37′ 52′′	73° 4′ 25′′	57	valley	-4.3 (10/1/2024)	29 (10/17/2022)	-2 (01/11/2021)	36.1 (01/22/2024)
El Tepual, Puerto Montt Ad.	41° 27′ 51′′	73° 6′ 45′′	90	valley	-2.3 (10/3/1994)	31.8 (10/25/2021)	0.8 (01/10/2019)	35.4 (01/31/2024)
Estación Mocopulli Ad.	42° 21′ 48′′	73° 43′ 54′′	174	coastal	-1.7 (10/1/2019)	22.9 (10/17/2022)	1.8 (01/17/2022)	28.2 (01/26/2017)
Estación Futaleufú Ad.	43° 11′ 20′′	71° 51′ 8′′	350	valley	-5.1 (10/14/2021)	28.2 (10/31/2020)	-0.4 (01/11/2021)	36.4 (01/22/2024)
Alto Palena	43° 37′ 42′′	71° 48′ 1′′	256	foothills	-4.9 (10/31/2022)	30.4 (10/19/2021)	-0.4 (01/11/2021)	37 (01/22/2024)
Estación Melinka Ad.	43° 54′ 52′′	73° 44′ 22′′	11	coastal	3.4 (10/29/2023)	22.3 (10/17/2022)	5.7 (01/11/2021)	28.3 (01/22/2024)
Estación Puerto Aysén Ad.	45° 24′ 58′′	72° 41′ 38′′	10	valley	-3.2 (10/3/1991)	26 (10/17/2022)	0.7 (01/16/2022)	34.9 (01/27/2022)
Teniente Vidal Ad.	45° 35′ 27′′	72° 6′ 8′′	299	valley	-7.3 (10/3/2020)	27.5 (10/31/2021)	0.0 (01/16/2022)	35.6 (01/27/1975)
Estación Balmaceda Ad.	45° 55′ 6′′	71° 41′ 40′′	517	valley	-9.1 (10/1/2024)	26.2 (10/22/2024)	-5.9 (01/17/2022)	33.2 (01/24/2008)

Figure S1

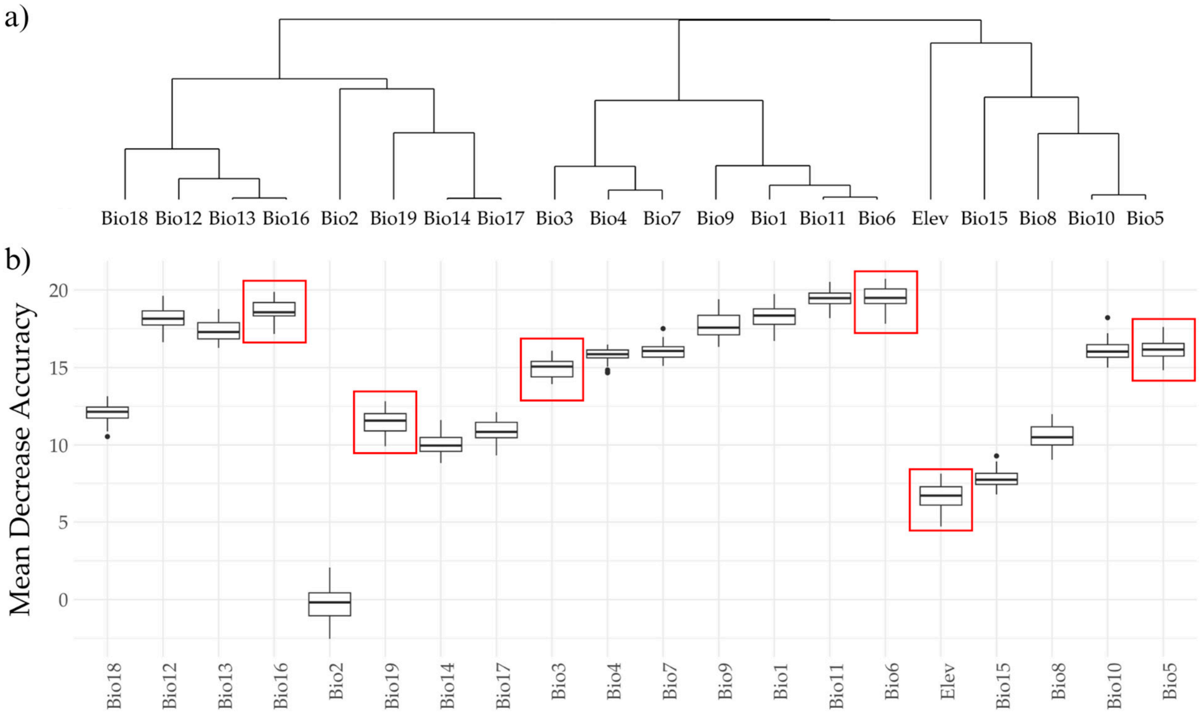

Figure S1: Climatic variables and filtering for modelling. a) UPGMA clustering based on the Pearson correlation among 19 bioclimatic variables and elevation data from WorldClim. b) Boxplot of the Mean Decrease Accuracy, calculated from 30 replicates of a Random Forest model that included the same selected variables. The red boxes highlight the variables selected within each cluster. Bioclimatic variables are: **Bio18**, Precipitation of the wettest quarter; **Bio12** Annual precipitation; **Bio13**, Precipitation of the wettest month; **Bio16**, Number of days with >10 mm precipitation; **Bio2**, Mean diurnal temperature range; **Bio19**, Precipitation of the driest quarter; **Bio14**, Precipitation of the driest month; **Bio17**, Number of days with >20 mm precipitation; **Bio3**, Isothermality (bio2 / bio7) * 100; **Bio4**, Temperature seasonality (standard deviation * 100); **Bio7**, Mean temperature of the warmest quarter; **Bio9**, Mean temperature of the driest quarter; **Bio11**, Mean temperature of the coldest quarter; **Bio6**, Min temperature of the coldest month; **Elev**, elevation; **Bio15**, Number of days with >1 mm precipitation; **Bio8**, Mean temperature of the coldest quarter; **Bio10**, Mean temperature of the wettest quarter; **Bio5**, Max temperature of the warmest month; **Bio1**, Annual mean temperature.

Figure S2

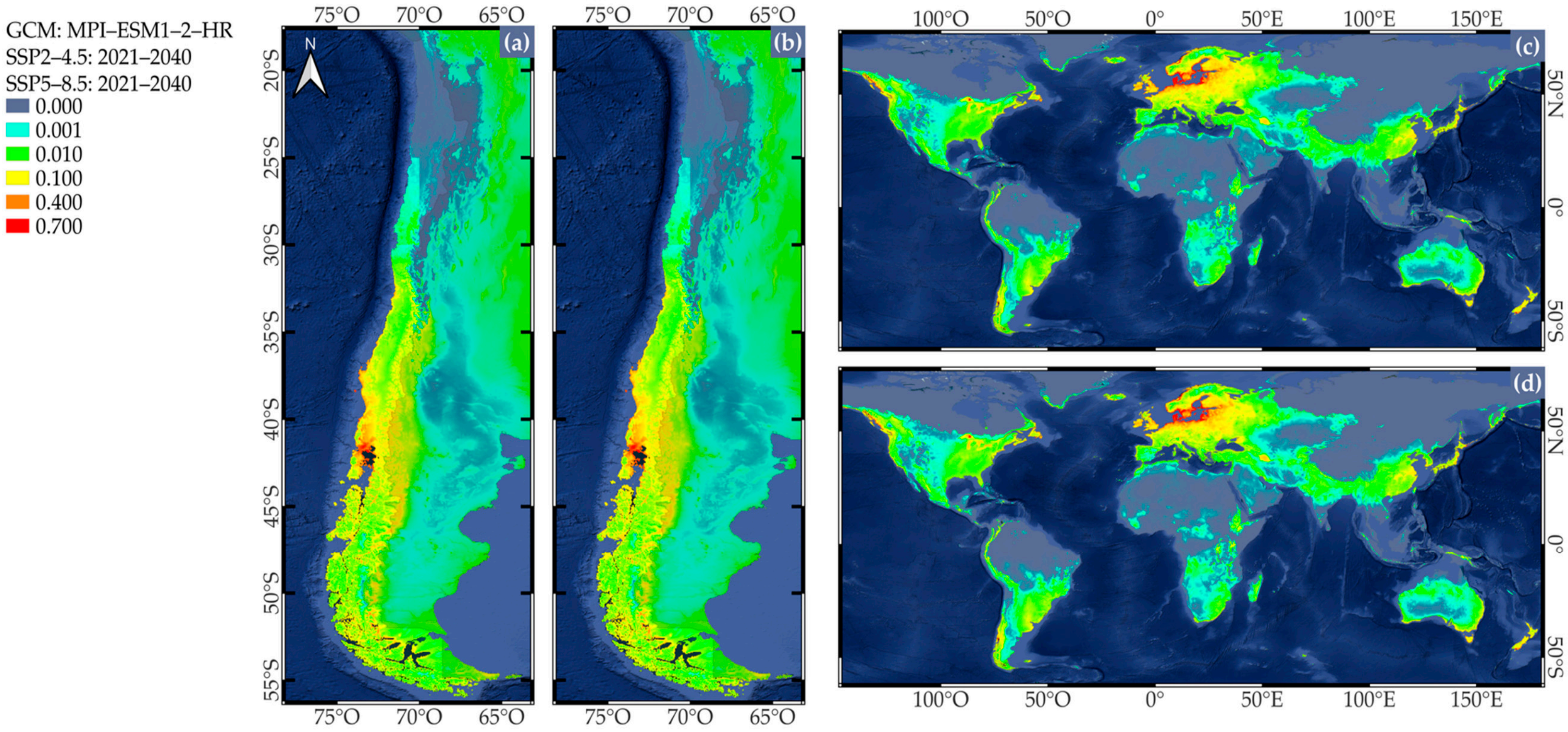

Figure S2. Projected habitat suitability for *Fragaria × ananassa* based on selected BIO variables from WorldClim, using the GCM: MPI-ESM1-2HR, for a) Chile under the SSP2-4.5 scenario for the 2021–2040 period, b) Chile under the SSP5-8.5 scenario for the same period, while c) and d) show Global projections under the same scenarios, respectively. The color gradient indicates habitat suitability, with warmer colors representing higher suitability.

Figure S3

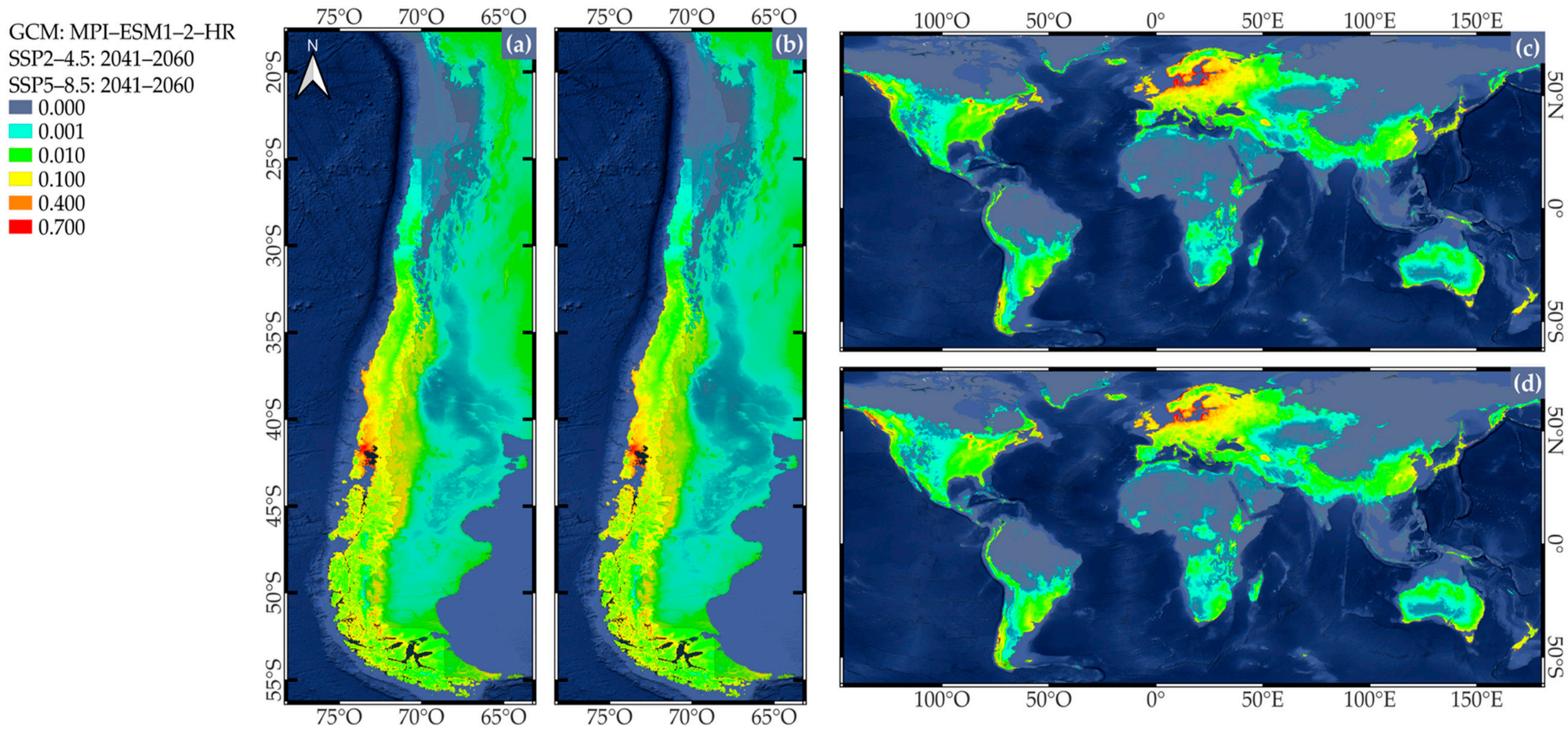

Figure S3. Projected habitat suitability for *Fragaria × ananassa* based on selected bioclimatic variables from WorldClim, using the GCM: MPI-ESM1-2HR, for a) Chile under the SSP2-4.5 scenario for the 2041–2060 period, b) Chile under the SSP5-8.5 scenario for the same period, while c) and d) show Global projections under the same scenarios respectively. The color gradient indicates habitat suitability, with warmer colors representing higher suitability.
